# Unusual presentation of plasmablastic lymphoma involving ovarian mature cystic teratoma: a case report

**DOI:** 10.1186/s13000-017-0672-x

**Published:** 2017-11-29

**Authors:** Ita Hadžisejdić, Emina Babarović, Luka Vranić, Antica Duletić Načinović, Ksenija Lučin, Maja Krašević, Nives Jonjić

**Affiliations:** 1 Department of Pathology, Faculty of Medicine, University of Rijeka, Braće Branchetta 20, 51000 Rijeka, Croatia; 2 Faculty of Medicine, University of Rijeka, Braće Branchetta 20, 51000 Rijeka, Croatia; 3 Department of Hematology, Rijeka University Hospital Centre, Krešimirova 42, 51000 Rijeka, Croatia

**Keywords:** Plasmablastic lymphoma, Ovarian mature cystic teratoma, Immune competent patient

## Abstract

**Background:**

Plasmablastic lymphoma (PBL) is relatively new clinical entity described as a distinct subtype of diffuse large B-cell lymphoma (DLBCL). It is characterized by its aggressive nature and proliferation of large neoplastic cells resembling immunoblasts including cells with more obvious plasmacytic differentiation. In this case report, we describe an unexpected finding of PBL associated with a mature cystic teratoma of the ovary in a young immune competent woman.

**Case presentation:**

A 19-year old woman was admitted to the hospital with generalized lymphadenopathy, a pelvic tumor mass measuring 35 × 30 cm and a 4 cm lump in her right breast. She underwent a right salpingo-oophorectomy, lymphadenectomy, splenectomy, omentectomy, and a right breast lumpectomy. On macroscopic examination the right ovary was replaced by a thick-walled multilocular cystic tumor. Upon incision, the cysts were filled with thick, greasy sebaceous material and hair and there were several solid nodules within the cyst walls. Histological examination revealed a mature cystic teratoma and malignant non-Hodgkin lymphoma (NHL) within the solid nodules. Tumor tissue from the right breast, spleen and lymph nodes, all had the same histological, NHL morphology. After extensive immunostaining, a diagnosis of PBL was made. Following surgery, the patient was treated with different chemotherapy regimens, without any significant regression of the disease, and died of multiple organ failure.

**Conclusions:**

Primary NHL of the ovary is relatively rare occurrence while secondary involvement by lymphoma is much more common. PBL is a rare lymphoma, primarily reported in the jaw and oral mucosa, but also documented in extra-oral sites. To the best of our knowledge, this is the first case described in a mature ovarian cystic teratoma. Although the patient was HIV-negative and immune competent, she had progressive disease and died despite aggressive chemotherapy 11 months after the initial diagnosis.

**Electronic supplementary material:**

The online version of this article (10.1186/s13000-017-0672-x) contains supplementary material, which is available to authorized users.

## Background

Plasmablastic lymphoma (PBL) is a rare and aggressive variant of NHL with diffuse proliferation of large neoplastic cells with cytomorphological features that resemble B immunoblasts, but with the immunophenotype of the plasma cells [1]. In the most cases, this neoplasm occurs in the oral cavity of HIV-positive patients. Other immunodeficiency states such as iatrogenic immune suppression in treatment of autoimmune diseases or chemotherapy are also associated with this diagnosis [1, 2]. It is primarily an adult disease that occurs most frequently around 50 years of age, affecting men more often than women. Although, the majority of patients are adults, it has also been reported in the pediatric age group [3]. Involvement of extra-oral sites, such as the orbit, sinonasal cavity, gastrointestinal tract, soft tissues, liver, retroperitoneal region and bone can also be found, especially in PBLs that are not associated with HIV infection [3]. The PBL cells are usually positive for a plasma cell phenotype including CD138, CD38, CD79a, IRF4/MUM1, cytoplasmic immunoglobulins (most frequently IgG) and either kappa or lambda light chain with a high Ki-67 proliferation index (usually ≥80%) [2, 3]. Approximately 70% of cases express EBV-encoded RNA (EBER), which is the most sensitive methodology for detecting EBV infection within the malignant cells [2]. Both HIV-positive and HIV-negative patients are at advanced clinical stage (Ann-Arbour III or IV) on presentation. This rare clinical entity represents a diagnostic challenge due to its specific morphological and immunohistochemical resemblance to lymphomas with plasmablastic differentiation, more precisely plasmablastic or anaplastic multiple myleoma. Furthermore, with its aggressive clinical course and almost 100% rate of death within the first year of diagnosis, it also remains a therapeutic challenge. This report presents a very unusual case of PBL in an immune competent, HIV-negative, 19-year old woman, that was detected within the walls of an ovarian cystic teratoma occurring as a part of systemic disease with involvement of the breast, spleen and lymph nodes.

## Case presentation

A 19-year old female was referred to the Department of Internal Medicine for workup of anemia. She complained of having fever (up to 38 °C), night sweats, dyspnea, dry cough, and fatigue. Symptoms had been present for 4 weeks and had not improved after a course of antibiotics prescribed by her family physician. On physical examination, a palpable painless mass was noticed in her right abdomen, with enlarged inguinal lymph nodes as well as a palpable lump in the right breast with ipsilateral axillary lymphadenopathy. Abdominal ultrasound (US) examination revealed a multiloculated cystic mass with solid areas measuring 35 × 30 cm in her pelvis. The US examination also showed an enlarged spleen with enlarged para-aortal lymph nodes. Subsequently, a thoracic, abdominal and pelvic multi-slice computed tomography scan (MSCT) was performed and showed a well circumscribed, solid nodule, 4 cm in diameter, in the upper outer quadrant of right breast with enlarged ipsilateral axillary lymph nodes, along with generalized abdominal lymphadenopathy, an enlarged spleen with diffuse multiple nodal masses and a complex right pelvic tumor mass with amorphous calcification similar to tooth formation. Based on the MSCT scan, a tentative diagnosis of an immature teratoma was made. The patient proceeded to surgery for a unilateral right salpingo-oophorectomy, lymphadenectomy, splenectomy, omentectomy and right breast lumpectomy. Intraoperative peritoneal washing revealed a poorly differentiated malignant tumor on cytology.

**Fig. 1:**
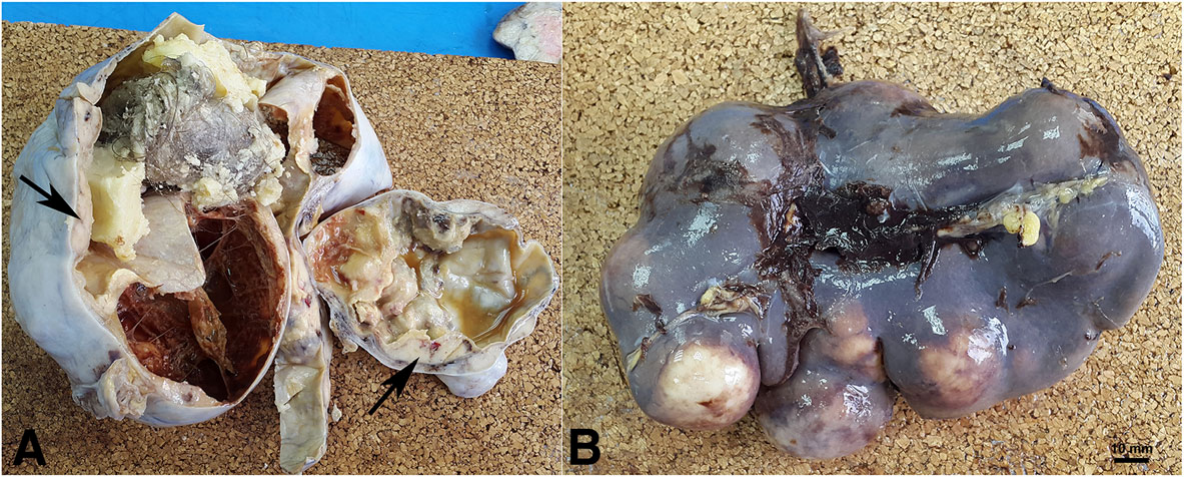
Macroscopic image of multiloculated ovarian cyst and spleen. **a** The right ovary was replaced by a cystic tumor (mature teratoma) with multiple solid nodules of PBL within the cyst’s walls (arrows). **b** Spleen with multiple PBL nodules

**Fig. 2:**
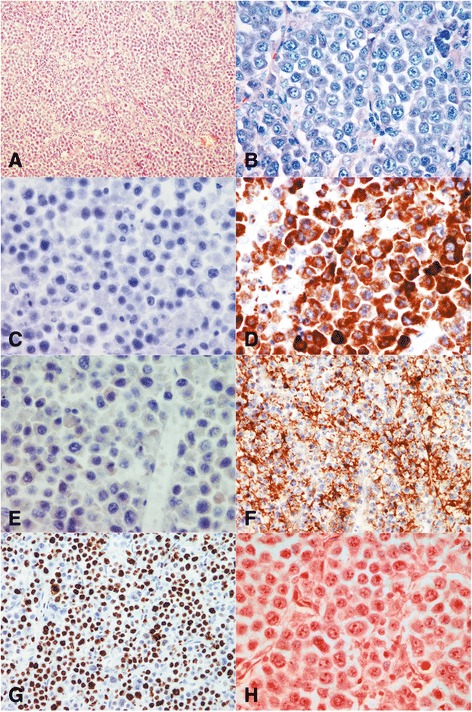
Histology and immunohistochemisty of plasmablastic lymphoma. **a** Haematoxylin and eosin section shows monotonous, lymphoid cells with “starry sky” pattern (magnification ×100). **b** Giemsa staining highlights the plasmablastic appearance with eccentrically positioned nuclei, prominent nucleoli and abundant basophilic cytoplasm (magnification ×400). **c**-**f** Atypical lymphoid cells were negative for CD20 (**c**) and CD30 (**e**), while they were positive for CD79a (**d**) and CD138 (**f**) (immunoperoxidase stain; **c**, **d** and **e** magnification × 400; **f** magnification ×200). **g** Nuclear proliferation rate, assessed by Ki-67 was approximately 80% (immunoperoxidase stain; magnification × 200). **h** EBV encoded RNA (EBER) was negative in the nuclei of the atypical cells (magnification ×400)

On macroscopic examination, the right ovary was replaced by a cystic mass with a glistening surface, measuring 17 cm in diameter (Fig. 1a). On incision, the cyst was partly filled with fatty material and hair. The wall of the cyst was thickened with solid, grayish nodules. The spleen was diffusely infiltrated with grayish nodes as well, measuring up to 4.5 cm in diameter (Fig. 1b). There was a lobulated, well circumscribed solid, grayish nodule identified on lumpectomy of the breast. On histological examination, the cavity of the cyst was lined by skin with dermal appendages as well as respiratory type of epithelium, smooth muscle, mature adipose and bone tissue. This morphology was in agreement with a diagnosis of mature cystic teratoma of the ovary. Solid nodules within cyst walls were tumor tissue, with a diffuse growth pattern, composed of large polymorphic cells with vesicular nuclei, a high nuclear-cytoplasmic ratio, and one or more prominent nucleoli. Some cells had eccentrically positioned nuclei with a cytoplasmic halo resembling plasmablasts. The tumor tissue from the right breast and spleen had the same histological appearance. An extensive immunohistochemical evaluation was performed. Tumor cells were negative for panCK, AFP, PLAP, CD117, D2-40, CD30, MyoD1, HMB45, desmin and CD99, but positive for LCA, EMA and vimentin. Therefore, a diagnosis of NHL was made. Subsequent immunohistochemistry showed tumor cells to be CD79a, PAX5, MUM-1, CD38, CD138 positive and CD20, CD10, Bcl-6, ALK, cyclin D1, CD56 negative. Ki-67 was high, around 80% and EBER in situ hybridization was negative (Fig. 2). According to the morphological and immunohistochemical findings a diagnosis of PBL was made. Based on the clinical findings, the patient was staged, Ann-Arbour IV PBL. Laboratory data showed anemia, normal platelet and leucocyte counts with elevated lactate dehydrogenase (LDH), aspartate aminotransferase (AST) and C-reactive protein (CRP). Serum alpha-1-fetoprotein and human chorionic gonadotropin were normal while carbohydrate antigen 125 was increased. Urine analysis and renal function were within normal limits. Serum immunoglobulin levels were within normal limits, without evidence of monoclonal gammopathy and serology was negative for HIV, HBV and HCV. The patient was transferred to the Hematology ward and treated with dose-adjusted etoposide, prednisone, vincristine, cyclophosphamide, and doxorubicin (DA-EPOCH) as well as intra-thecal EPOCH with each cycle [2, 4]. After 4 cycles of chemotherapy, the patient subjectively felt better however evaluation with positron emission tomography and computed tomography scans (PET-CT) showed high activity of disease. An autologous peripheral blood stem cell transplantation was planned and 1 cycle of salvage chemotherapy was given according to DHAP protocol (cisplatin, cytarabine and dexamethasone). Unfortunately, her underlying disease continued to progress and 4 cycles of alternating modified CODOX-M and IVAC chemotherapy (cyclophosphamide, vincristine, doxorubicin, high-dosemethotrexate/ifosfamide, etoposide, and high-dosecytarabine) were given. Autologous stem cell transplantation could no longer be performed due to patient’s clinical deterioration. After the fourth cycle of chemotherapy, PET-CT scan was performed again, and revealed profound disease progression. The patient also had developed treatment complications, such as secondary anemia, thrombocytopenia, sepsis, enterocolitis, ileus, pleural effusions and ascites. Due to the worsening of the patient’s physical condition, her goals of care were changed and only supportive therapy was given. The patient died of multiple organ failure approximately 11 months after initial diagnosis (Additional file 1).

**Fig. 3:**
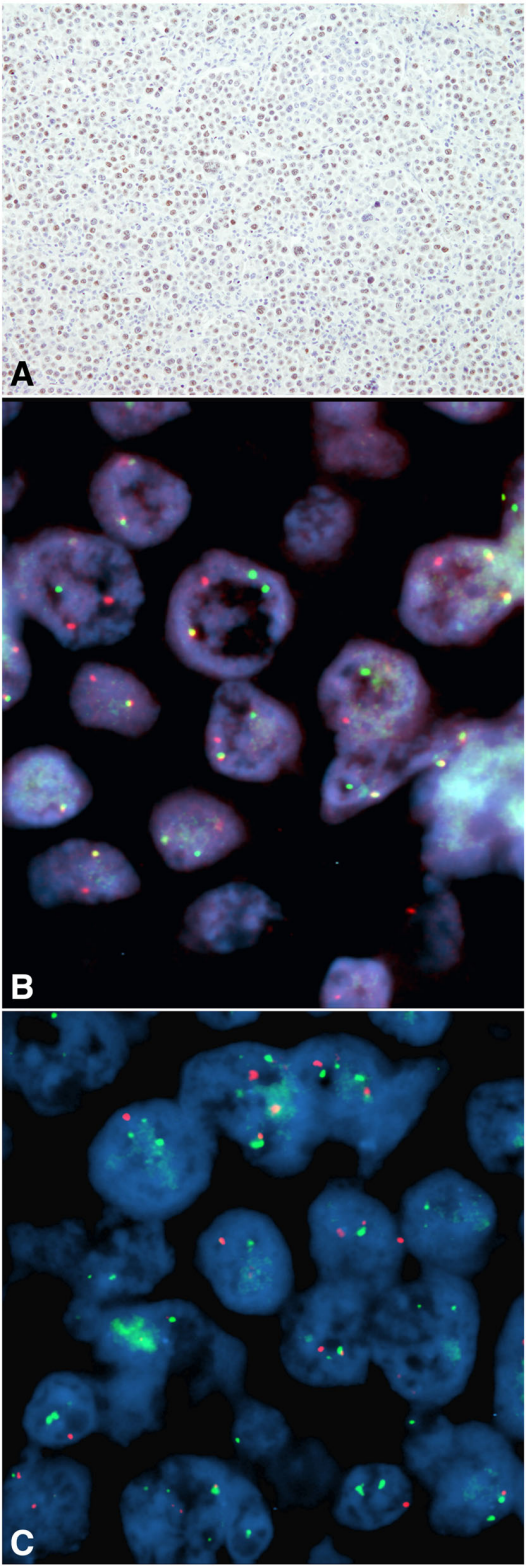
MYC immunohistochemistry and fluorescent in situ hybridization (FISH). **a** Large atypical plasmablasts were c-MYC positive in >40% of cells. **b** FISH analysis using Vysis *MYC* Break Apart FISH Probe Kit revealed 8q24 rearrangement within tumour cells (cells without rearrangement should have 2 yellow fusion signals; magnification × 1000). **c** FISH analysis using Vysis LSI *IGH/MYC*, CEP 8 Tri-color Dual Fusion Translocation Probe is negative for t(8,14), without yellow signals specific for *IGH/MYC* translocation (normal 2 green (*IgH*) and 2 red (*MYC*, 8q24) signals); magnification × 1000

## Discussion

PBL was originally reported exclusively in the jaw and oral mucosa of male, predominantly HIV-positive, patients [5]. It may be present in patients who are HIV-negative, but otherwise immune compromised such as patients with solid organ or bone marrow transplantation or those being treated for autoimmune disorders [6]. Cases of PBL occurring in elderly, immune competent patients have been documented and increasingly described in extra-oral locations [1, 6, 7]. Extra-oral PBL has been reported in various locations; however to the best of our knowledge, PBL was never described within ovarian cystic teratoma. This case is unusual as it presents the first described case of PBL in a 19-year old, immune competent patient, within the walls of a mature ovarian cystic teratoma, with concurrent involvement of spleen, lymph nodes and breast tissue. Primary NHL of the ovary is a relatively rare occurrence while secondary involvement is much more common [8]. Descriptions of NHL arising in mature cystic teratoma are even more rare. There have only been a few reports describing DLBC and follicular lymphoma arising in a mature cystic teratoma [9–11]. Primary ovarian lymphoma accounts for 0.5% of extranodal NHL and 1.5% of primary ovarian cancers [12]. The existence of primary ovarian NHL is a question of debate by some authors because there is no pre-existing lymphoid tissue in the ovaries for lymphoma to develop. However, some studies have identified small number of lymphocytes around blood vessels at the hilum and within epithelial structures of teratomas, supporting the hypothesis for possible development of primary ovarian lymphoma [13, 14] from this lymphoid tissue. Also, due to varying definitions, the existence of primary ovarian lymphoma remains debatable. Fox et al. proposed the following diagnostic criteria for a primary ovarian lymphoma: 1. The lymphoma should be confined to the ovary or at the maximum, to the adjacent group of lymph nodes draining the organ involved or if there has been direct infiltration of adjacent structures while full investigation fails to reveal evidence of lymphoma elsewhere; 2. The peripheral blood and bone marrow should not contain any abnormal cells; 3. If further lymphomatous lesions occur at sites remote from the ovary, then at least several months should have elapsed between the appearance of the ovarian and extra-ovarian lesions [15]. According to this definition, our patient’s PBL was disseminated, secondary disease with concurrent involvement of the ovarian mature cystic teratoma. Also, our patient presented with B symptoms as a main complaint, which is more suggestive of secondary involvement by PBL because the majority of patients with primary ovarian lymphomas present with abdominal or pelvic pain as their main complaint. Possible origin from a teratoma with differentiation along hematopoietic cell lines could be considered, but it is less likely and difficult or impossible to prove.

EBV plays an important role in the tumorigenesis of PBL with expression of EBER by in situ hybridization (ISH) in 82% and 46% of the HIV-positive and HIV-negative cases, respectively [3]. The presence of plasmablasts is noted in reactive processes, associated with viral infections, but nevertheless pathogenesis of PBL is poorly understood.

In addition to EBV infection, recent studies have identified the presence of *MYC* gene rearrangements as important pathogenic mechanisms [16, 17]. Using ISH, Valera et al., examined a larger population of PBL patients and found *MYC* rearrangement in 49% of the cases [18]. In PBL, *MYC* deregulation, mediated by translocation or amplification allows MYC to overcome the regulatory effects of BCL-6 or BLIMP-1. BCL-6 is a repressor of *MYC* in the germinal center B cells, whereas BLIMP-1 is a repressor of *MYC* in terminally differentiated B cells [2]. According to one theory, *MYC* translocation may lead to the plasmablastic morphology and create a more aggressive disease state [3, 19]. Also, it has been observed that *MYC* rearrangements are more often seen in EBV-positive compared to EBV-negative tumors [18]. Therefore, we also performed c-MYC immunohistochemistry which showed strong protein expression in >40% of tumor cells (Fig. 3). Furthermore, to examine *c-MYC* gene status, fluorescent in situ hybridization (FISH) was performed using Vysis *MYC* Break Apart FISH Probe Kit (Abbott Molecular, IL, USA) and Vysis LSI *IGH/MYC*, CEP 8 Tri-color Dual Fusion Translocation Probe (Abbott Molecular, IL, USA). We found 8q24 rearrangement within tumor cells but we did not find specified t (8,14) *IGH/MYC* translocation (Fig. 3).

The present case was negative for HIV and EBV infection was not detected by ISH. Some studies revealed that EBV infection was detected in only 17% of HIV-negative PBL cases, suggesting that EBV is not a distinct contributor in the pathogenesis of PBL in patients without HIV infection [20, 21].

The minimum morphologic criteria, required to diagnose PBL, are monomorphic cellular proliferation of plasmablasts, with either centrally or eccentrically placed nuclei, with high nuclear-cytoplasmic rate, a moderate amount of eosinophilic cytoplasm, a high mitotic index and the absence of plasma cells in the background [22, 23]. High mitotic activity found in all PBLs as determined by Ki-67 proliferation index, is consistent with our case. PBL should also be differentiated from other malignant diseases including DLBCL, poorly differentiated carcinomas, melanoma, gastrointestinal stromal tumors, Burkitt’s lymphoma and anaplastic large cell lymphoma. However, the main differential diagnosis of PBL is plasmablastic or anaplastic multiple myeloma. In practice the distinction between these frequently depends on clinical correlation. HIV infection, immune suppression and EBER positivity favor PBL diagnosis, while the presence of monoclonal paraproteinemia, lytic bone lesions, hypercalcemia and renal dysfunction favors a diagnosis of myeloma. Also high Ki-67 proliferation index and CD56 positivity favors diagnosis of PBL over myeloma [6].

PBL is a highly aggressive disease with the majority of patients dying within the first year after initial presentation. A systemic review of HIV-positive patients with PBL showed a median overall survival (OS) of 15 months versus 9 months in HIV-negative patients [24]. Furthermore, very high Ki-67 index and *MYC* gene rearrangements, have been shown to be associated with shorter OS in patients with PBL [2, 21]. Also extra-oral PBL are more frequently disseminated at the time of diagnosis (57% of patients are at stage IV) [21]. The prognostic value of EBV related antigens expression in PBL cases is unclear. Some studies have shown that EBV is not associated with outcome in HIV-associated PBL while other studies have shown that EBV infection correlated with better outcome in immune competent patients [2]. In our case, the patient’s immune competent, HIV-negative status, EBER negativity, extra-oral location, high Ki-67 proliferation index and *MYC* rearrangement were all predictors of the aggressive clinical course and short survival.

PBL remains difficult to diagnose and to treat. A standard therapeutic approach for patients with PBL has not been established. In particular, the use of cyclophosphamide, doxorubicin, vincristine and prednisone (CHOP) is considered inadequate therapy, and current guidelines recommend more intensive treatments [2]. Such treatments include infusional etoposide, vincristine and doxorubicin with bolus cyclophosphamide and prednisone (EPOCH), cyclophosphamide, vincristine, doxorubicin, methotrexate alternating with ifosfamide, etoposide and cytarabine (CODOX-M/IVAC) [25], or hyperfractionated cyclophosphamide, vincristine, doxorubicin, and dexamethasone alternating with methotrexate and cytarabine (hyper-CVAD) [26]. More recently, the role of stem cell transplantation (SCT) in patients with PBL has been assessed [27]. It appears patients with PBL with chemotherapy-sensitive disease might benefit from autologous SCT in the first remission therefore, it seems rational to explore the use of autologous SCT earlier in the disease course [27]. Some studies have reported that bortezomib (proteasome inhibitor) alone or in combination with chemotherapy may have an antitumor effect in PBL considering it has many morphologic and immunophenotypic similarities with myeloma [28]. In our case, the patient received 4 cycles of DA-EPOCH, followed by 1 cycle of salvage DHAP and 4 cycles of alternating modified CODOX-M/IVAC chemotherapy. Unfortunately autologous SCT could not be performed due to the patient’s worsening clinical status.

## Conclusions

In conclusion, we report a peculiar, extra-oral PBL case detected within a mature cystic teratoma in an HIV-negative, immune competent patient. To the best of our knowledge, this is the first case of PBL described in a mature ovarian cystic teratoma. Diagnosis of PBL is challenging, especially when it arises in younger, HIV-negative, immune competent, female patients without EBER positivity. Currently there is no definitive treatment regimen capable of providing curative results, therefore until standardized chemotherapy is identified, management of patients with PBL should be on case to case basis [29].

## Additional file

Additional file 1: Timeline table Hadžisejdić et al. case report. (DOC 39 kb)

## Abbreviations

AST: Aspartate aminotransferase
CD: Cluster of differentiation
CRP: C-reactive protein
DLCBL: Diffuse large B-cell lymphoma
EBER: EBV-encoded RNA
EBV: Ebstein-Barr virus
FISH: fluorescent in situ hybridization
HBV: Hepatitis B virus
HCV: Hepatitis C virus
HIV: Human immunodeficiency virus
ISH: In situ hybridisation
LDH: Lactate dehydrogenase
MSCT: Multi-slice computed tomography
NHL: Non-Hodgkin lymphoma
OS: Overall survival
PBL: Plasmablastic lymphoma
PET-CT: Positron emission tomography and computed tomography
SCT: Steam cell transplantation
US: Ultrasound
