# Supplementary material for: Unusual presentation of plasmablastic lymphoma involving ovarian mature cystic teratoma: a case report
Source: Diagn Pathol. 2017 Nov 29;12:83. doi: 10.1186/s13000-017-0672-x (PMC5707791; doi:10.1186/s13000-017-0672-x)
Supplement: Additional file 1: — Timeline table Hadžisejdić et al. case report. (DOC 39 kb) [file 13000_2017_672_MOESM1_ESM.doc]

Timeline table Hadžisejdić et al case report

| Dates | Past Medical History and Interventions | | |
| --- | --- | --- | --- |
| **At presentation** | Four weeks earlier-night sweats, dry cough, fatigue, shortness of breath and increased temperature, up to 38˚C  Family physician prescribed antibiotics-no improvement  Family physician performed routine blood and urine workup- anemia and increased CRP  Subsequently a 19 year old female was referred to the hospital, at Department of Internal Medicine for anemia work up | | |
| Dates | Summaries from initial and Follow up Visits | Diagnostics Testing | Interventions |
| **A month after initial presentation** | Palpable painless mass in her right abdomen, enlarged inguinal lymph nodes and palpable lump in the right breast with ipsilateral axillary lymphadenopathy | Abdominal ultrasound - pelvic multiloculated cystic tumor mass, increased spleen and enlarged paraaortal lymph nodes | Transferred to gynecological ward due to suspected gynecological malignancy |
|  | Right breast - palpable nodule | Breast ultrasound | Cytological punction |
|  |  | Tumor antigens from the blood-increased Ca 125 |  |
|  |  | Thoracic, abdominal and pelvic MSCT-well circumscribed solid nodule, measuring 4 cm in diameter in the upper outer quadrant of the right breast with enlarged ipsilateral axillary lymph nodes, along with generalized abdominal lymphadenopathy, enlarged spleen, diffusely infiltrated with multiple nodal masses and complex right pelvic tumor mass with amorphous calcification which was similar to tooth formation |  |
| **A month after initial presentation** |  |  | Surgery- right salpingo-oophorectomy, spleenectomy, right breast lumpectomy, lymphadenectomy and omentectomy with peritoneal washings |
|  |  | Cytology- intraoperative peritoneal washing revealed poorly differentiated malignant tumor |  |
| **2 months after initial presentation** |  | Pathology-diagnosis of non-Hodgkin lymphoma, plasmablastic lymphoma (PBL) | Staged IV PBL |
|  |  |  | Chemotherapy-DA-EPOCH protocol (4 cycles) |
| **6 months after initial presentation** | Subjectively better | PET-CT scans showed high activity of disease | One cycle of salvage chemotherapy - DHAP protocol |
| **7 months after initial presentation** | Patient was feeling worse, weight loss |  | Autologous peripheral blood stem cell transplantation was planned |
|  | Diseases progression |  | Four cycles of alternating modified CODOX-M and IVAC chemotherapy was given |
| **9 months after initial presentation** | Subjectively better |  |  |
| **11 months after initial presentation** |  | PET-CT scans showed profound disease progression |  |
|  | Patient developed treatment complications- secondary anemia, thrombocytopenia, sepsis, enterocolitis, ileus, pleural effusion and ascites. |  | Only supportive therapy was given |
| **12 months after initial presentation** | Patient died due to multiple organ failure |  |  |
